# Activated B Lymphocyte Inhibited the Osteoblastogenesis of Bone Mesenchymal Stem Cells by Notch Signaling

**DOI:** 10.1155/2019/8150123

**Published:** 2019-06-02

**Authors:** Mengxue Pan, Wei Hong, Ye Yao, Xiaoxue Gao, Yi Zhou, Guoxiang Fu, Yuanchuang Li, Qiang Guo, Xinxin Rao, Peiyuan Tang, Shengzhi Chen, Weifang Jin, Guoqiang Hua, Jianjun Gao, Xiaoya Xu

**Affiliations:** ^1^Department of Radiation Biology, Institute of Radiation Medicine, Fudan University, No. 2094 Xie-Tu Rd. Building 1, Shanghai 200032, China; ^2^Department of Geriatrics, Huadong Hospital, Research Center on Aging and Medicine, Fudan University, Shanghai 200040, China; ^3^Department of Bone Metabolism, Shanghai Key Laboratory of Clinical Geriatric Medicine, Shanghai 200040, China; ^4^Department of Radiation Oncology, Fudan University Shanghai Cancer Center, Fudan University, Shanghai 200032, China

## Abstract

Estrogen is very important to the differentiation of B lymphocytes; B lymphopoiesis induced by OVX was supposedly involved in osteoporosis. But the effects of B lymphocytes on the osteogenic differentiation of bone mesenchymal stem cells (BMSCs) are not clear. In this study, we detected bone quality and bone loss in a trabecular bone by electronic universal material testing machine and microcomputed tomography (micro-CT) in OVX and splenectomized-ovariectomy (SPX-OVX) rats. Additionally, changes in lymphocytes (B lymphocyte, CD4^+^ and CD8^+^ T lymphocytes, and macrophages) in the bone marrow were analyzed by flow cytometry. The osteogenesis of BMSCs cocultured with normal and LPS-pretreated B lymphocytes was detected by BCIP/NBT and Alizarin red S staining. Measurement of the Notch2, Notch4, Hey1, Hey2, Hes1, and runt-related transcription factor 2 (Runx2) expression in BMSCs cocultured with B lymphocytes was done using real-time PCR. The effects of dexamethasone and DAPT (inhibitor of Notch signaling) on osteogenesis of BMSCs were detected by BCIP/NBT, Alizarin red S staining, and real-time PCR. Osteoporosis happened in OVX rats, more serious in SPX-OVX rats, B lymphocytes increased in OVX rats, and sharply higher in SPX-OVX rats. Osteoporosis did not happen in SPX rats which is still companied with a high increase of B lymphocytes. LPS-pretreated B lymphocytes suppressed the osteogenesis of BMSCs, but the normal B lymphocytes could not. The LPS-pretreated B lymphocytes upregulated the expression of Notch4, Hes1, and Hey2 and downregulated the expression of Runx2 in BMSCs. Dexamethasone and DAPT could downregulate the high expression of Notch4, Hes1, Hey2 and upregulate the low expression of Runx2 in BMSCs which cocultured with LPS treated B lymphocytes, the inhibited ALP and Alizarin red staining in BMSCs which cocultured with LPS treated B lymphocytes also partly restored.

## 1. Introduction

It has become clear that complex interactions underlie the relationship between the skeletal and immune systems. This is particularly true for the development of immune cells in the bone marrow as well as the functions of bone cells in skeletal homeostasis and pathologies. Estrogen deficiency caused by ovariectomy (OVX) results in a marked bone loss due to exceeded bone resorption by increased osteoclasts (OC), which are partly stimulated by the immune system [1]. Increase of T lymphopoiesis by OVX is detected in OVX mice [2, 3]; expanded T cells stimulate osteoclastogenesis by more cytokine production such as RANKL, TNFa, and IFN-gamma in OVX mice, which half of bone loss was attenuated by thymectomy [2]. The number of B lymphocytes in bone marrow increased after OVX, and these activated B lymphocytes expressed RANKL contributing to bone resorption [4, 5]. Changes in B lymphocyte populations in the blood of postmenopausal osteoporosis patients have been shown [6]. However, as one of the important lymphocytes in the immune system, the role of B lymphocytes in bone mesenchymal stem cells of bone loss induced by estrogen deficiency remains unknown. These experiments were designed to investigate the skeleton phenotypes in splenectomized OVX female rats and the effects of B lymphocytes on OVX-induced bone loss. Meanwhile, we detected the differentiation of BMSCs cocultured with B lymphocytes which were pretreated with LPS. We also investigated the effects of dexamethasone in the differentiation of BMSCs which were cocultured with B lymphocytes and the changes of the Notch signaling in BMSCs; then, we used the inhibitor of Notch signaling to investigate the differentiation and the expression of Notch signaling in BMSCs.

## 2. Materials and Methods

### 2.1. Animal Studies

Female Sprague-Dawley rats (Shanghai Lab Animal Resource Center, STCSM, Shanghai, China) were bilateral splenectomized (SPX), ovariectomized (OVX), splenectomized OVX (SPX-OVX), and sham-operated (Sham), respectively, at 6 months of age under anesthesia. The animals were treated with benzylpenicillin sodium (D1110226, NCPC, China) for three days. All rats were maintained in a virus- and parasite-free barrier facility and exposed to a 12 h/12 h light/dark cycle and allowed free access to water and commercial standard rodent chow (containing: calcium: 1.8%, phosphorus: 0.6-1.2%). Tissues were collected at 3 months after surgery for densitometry, histomorphometry, and flow cytometry studies, respectively. This study was approved by the ethical committee for animal experiments in Fudan University, and all efforts were made to minimize suffering.

### 2.2. Histological Analyses of Bone

Bone mineral density (BMD) of either the femur or the lumbar (L1-5) was determined by dual-energy X-ray absorptiometry (DXA, Discovery A, Hologic Inc., Bedford, MA, USA) using an animal model at 3 months after surgery. The biomechanical quality was evaluated by the three-point bending test (femur) and compress test (L2), respectively, performed on an electronic universal material testing machine (INSTRON-5543, USA). For histomorphometry, the tissues were removed and fixed in PLP fixative (2% paraformaldehyde containing 0.075 M lysine and 0.01 M sodium periodate solution) 2 days at 5°C and processed histologically. Briefly, the distal end of the femurs was decalcified in EDTA glycerol solution for 28–30 days at 5°C. After paraffin embedding, 5 *μ*m sections were cut on a rotary microtome. The sections were stained with H&E, and the changes of bone trabecula were measured with bone volume over total volume (BV/TV, %).

### 2.3. Micro-CT Analysis

The tibiae and lumbar (L3) obtained from rats were dissected free of soft tissue, fixed overnight in 70% ethanol, and analyzed by micro-CT with a SkyScan-1176 scanner (Bruker microCT, Belgium) [7]. The trabecular bone region of interest (ROI) was drawn to include all cancellous bone in the whole area of 0.2 mm below the growth plate for trabecular bone mineral density (tBMD) analysis. The trabecular bone volume fraction (BV/TV), trabecular thickness (Tb.Th), number (Tb.N), and separation (Tb.Sp) were calculated on a 2 mm region of metaphysical spongiosa 0.2 mm below the growth plate.

### 2.4. Flow Cytometry

Bone marrow was collected 3 months after surgery. B lymphocytes, macrophages, and CD4^+^ and CD8^+^ T lymphocytes were detected by flow cytometry. The antibodies and protocol were previously described [7, 8].

### 2.5. Cell Culture and Coculture

BMSCs and B lymphocytes of the spleen from normal rats were collected according to the previous protocol [7]: (1) normal BMSCs cocultured for 3 days with different B lymphocytes density (0, 10^4^, 2 × 10^4^, 4 × 10^4^) for 3 days; (2) B lymphocytes treated with LPS (0, 1, 10, 100 *μ*mol/L) for 3 days, then collected cells and immediately cocultured with normal BMSCs for 3 days in the same B lymphocytes density (2 × 10^4^). ALP staining and Alizarin red staining were performed at 14 and 28 days.

LPS- (10 *μ*mol/L) treated B lymphocytes above were cocultured with normal BMSCs for 3 days and grouped into (1) control (BMSCs+without lymphocyte), lymphocyte (BMSCs+lymphocyte), DXM (BMSCs+dexamethasone (DXM, 10^−8^ M)), and DXM+lymphocyte (BMSCs+lymphocyte+DXM, 10^−8^ M); (2) control (BMSCs+without lymphocyte), DAPT (BMSCs+DAPT, 5 *μ*M), lymphocyte (BMSCs+lymphocyte), and lymphocyte+DAPT (BMSCs+lymphocyte+DAPT, 5 *μ*M). Some samples were used for real-time PCR; others were replaced with osteogenic differentiation medium; ALP staining and Alizarin red staining were performed at 14 and 28 days.

### 2.6. Quantitative Real-Time PCR

The real-time PCR was performed according to the previous protocol [7]. Primers with the following sequences were obtained from SBSgene (http://www.sbsgene.com) using a previously described protocol: Runx2, 5′-AGCCTCTTCAGCGCAGTGAC-3′ and 5′-CTGGTGCTCGGATCCCAA-3′ (132 bp, AF187319); Hey1, 5′-AGTGAGCTGGACGAGACCAT-3′ and 5′-CTGGGTACCAGCCTTCTCAG-3′ (197 bp, XM_017590595.1); Hey2, 5′-GATCTGCCAAGTTGGAAAAGG-3′ and 5′- TGTTGCCTGGAGCATCTTC-3′ (71 bp, NM_130417.1); Hes1, 5′-CAGAAAGTCATCAAAGCCTATCATG-3′ and 5′-TCAGTGTTTTCAGTTGGCTCAAA-3′ (80 bp, NM_024360.3); Notch4: 5′-CCTGGACAG CAATGCCAAGA-3′ and 5′-AGTCCAGCCCTCGTTACACACAC-3′ (147 bp, XM_017601710.1); Notch2: 5′-GACTGCCAATACTCGACCTC-3′ and 5′-TTCAGAAGTGAAGTCTCCAG-3′ (438 bp, NM_024358.1); and GAPDH, 5′-AAACCCATCACCATCTTCCA-3′ and 5′-GTGGTTCACACCCATCACAA-3′ (198 bp, DQ403053). The cDNA content was normalized by subtracting the cycle numbers of GAPDH from those of the target gene (*∆*Ct = Ct of target gene–Ct of GAPDH), and gene expression levels were calculated using the 2^–(∆Ct)^ method [9].

### 2.7. Statistical Analysis

Differences were determined by one-way ANOVA with Bonferroni post hoc testing or by paired or unpaired Student's *t*-test, as appropriate (GraphPad, Prism 6, version 6.0c). The results were expressed as the means ± standard derivations, and *p* < 0.05 was considered significant.

## 3. Results

### 3.1. The Bone Mass and Biomechanical Quality Changed in OVX and SPX-OVX Rats

**Figure 1:**
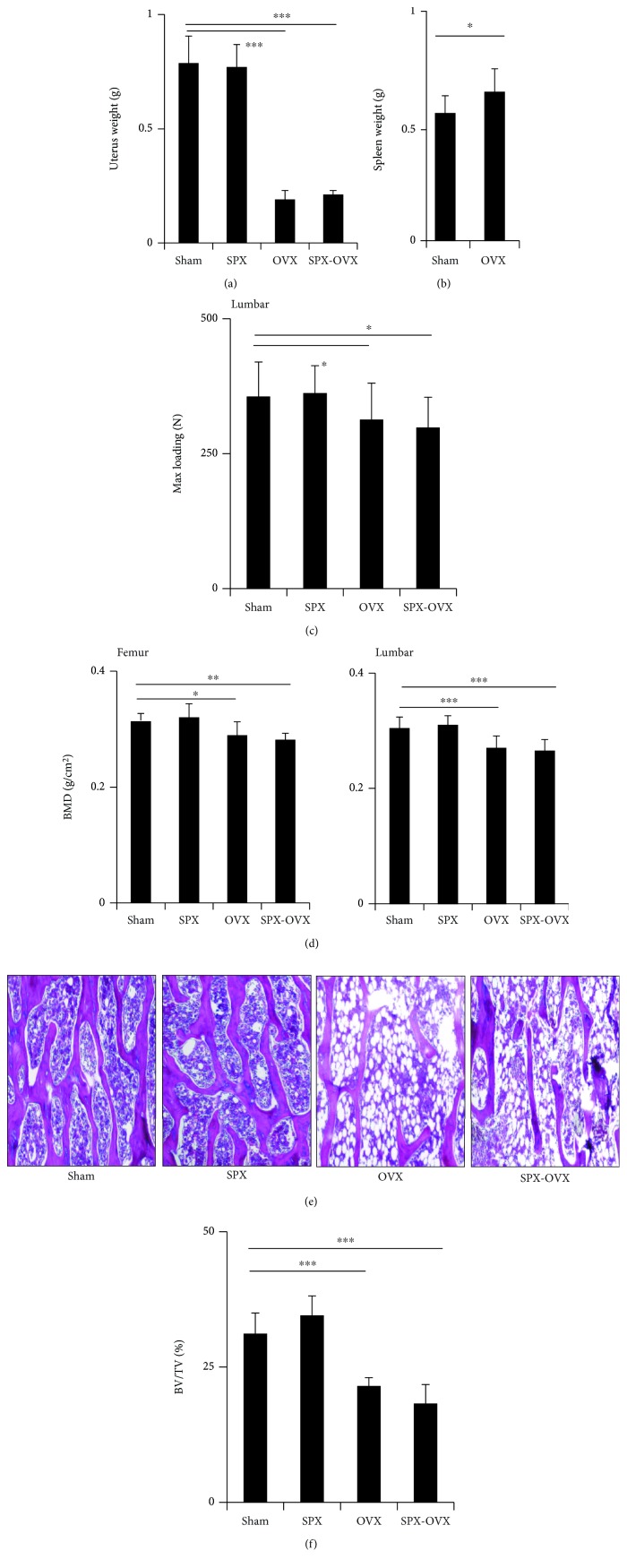
Effects on bone in OVX and SPX-OVX rats. (a) Uterus weight. (b) Spleen weight. (c) Max loading of the lumbar reduced in SPX and SPX-OVX. (d) BMD of the femur and lumbar decreased in SPX and SPX-OVX. (e) The HE staining showed decreased bone trabecula of the femur. (f) Bone volume of the femur decreased in SPX and SPX-OVX. Data are presented as the means ± standard deviations; ^∗^*p* < 0.05, ^∗∗^*p* < 0.01, and ^∗∗∗^*p* < 0.001 (*n* = 10/group).

Uterus weight from OVX and SPX-OVX rats decreased obviously while the spleen weight increased 3 months after surgery (Figures 1(a) and 1(b)). The max loading of the femur, as determined by the three-point bending test, was slight but not significant. The max loading of the lumbar as determined by the compress test was reduced by 13% (*p* < 0.05) and 15% (*p* < 0.05) in OVX and OVX-SPX, respectively, compared with Sham (Figure 1(c)). BMD of the femur and lumbar, as determined by dual-energy X-ray absorptiometry (DXA), was decreased by 6% (*p* < 0.05) and 19% (*p* < 0.001), respectively, in OVX rats compared with Sham but was decreased by 6.5% (*p* < 0.01) and by 19.8% (*p* < 0.001) in OVX-SPX rats compared with Sham (Figure 1(d)). After 3 months of surgery, the structure of trabecular bone was significantly altered in the OVX and OVX-SPX compared with Sham. The diminished trabecular bone volume decreased by 29% (*p* < 0.001) and 42% (*p* < 0.001), respectively, in OVX and OVX-SPX rats compared with Sham (Figures 1(e) and 1(f)).

### 3.2. Bone Microarchitecture Is Changed in OVX and SPX-OVX Rats

**Figure 2:**
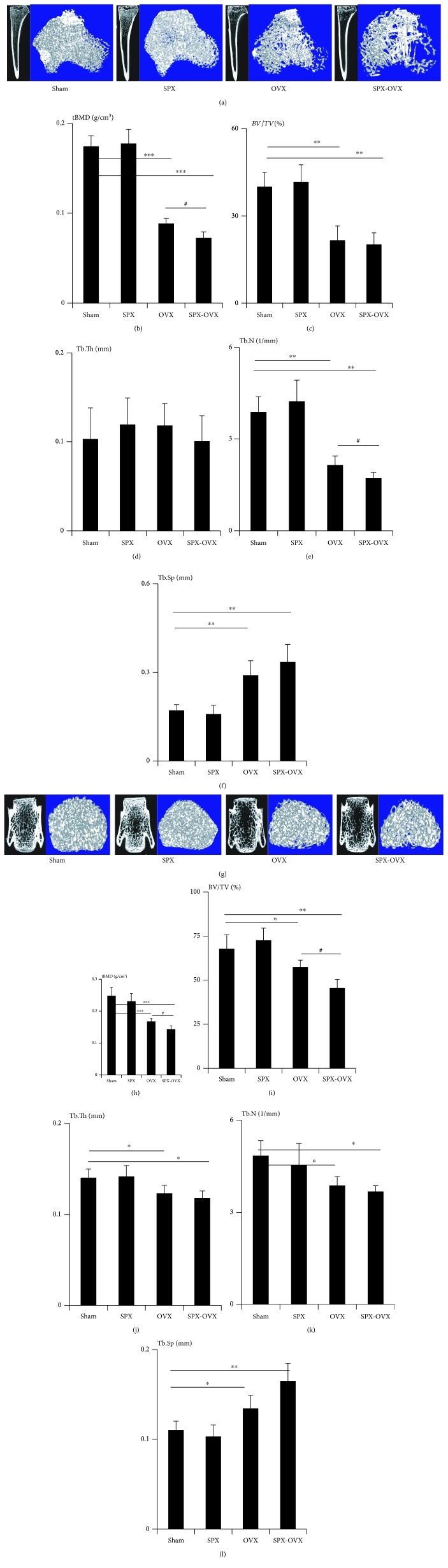
Effects on trabecular of the tibia and lumbar of OVX and SPX-OVX rats. (a, g) Representative reconstructed images of *μ*CT scans showing trabecular in the tibia and lumbar. (b) tBMD changed in the tibia. Differences in (c) BV/TV, (d) Tb.Th, (e) Tb.N, and (f) Tb.Sp in the tibia. (h) tBMD changed in the lumbar. Differences in (i) BV/TV, (j) Tb.Th, (k) Tb.N, and (l) Tb.Sp in the lumbar. Data are presented as the means ± standard deviations; ^∗^compared with Sham, ^∗^*p* < 0.05, ^∗∗^*p* < 0.01, and ^∗∗∗^*p* < 0.001; ^#^compared with OVX, ^#^*p* < 0.05 (*n* = 10/group).

We used *μ*CT to delineate a purely trabecular region of interest that showed changes in bone volume and microarchitectural structure. Three months after surgery, trabecula bone mineral density (tBMD) was reduced by 49.3% (*p* < 0.001) in the OVX tibia and by 58.5% (*p* < 0.001) in the SPX-OVX tibia relative to Sham, while further decreased by 18.1% (*p* < 0.05) in the SPX-OVX tibia relative to OVX (Figures 2(a) and 2(b)). The trabecular bone volume (BV/TV) was reduced by 46.1% (*p* < 0.01) in the OVX tibia and by 49.5% (*p* < 0.01) in the SPX-OVX tibia relative to Sham (Figure 2(c)). The Tb.Th of the tibia only changed slightly, and no significant differences were observed (Figure 2(d)). The Tb.N was reduced by 44.8% (*p* < 0.01) in the OVX tibia and by 56.0% (*p* < 0.01) in the SPX-OVX tibia relative to Sham (Figure 2(e)). The Tb.Sp increased by 69.5% (*p* < 0.01) in the OVX tibia and by 95.6% (*p* < 0.01) in the SPX-OVX tibia relative to Sham (Figure 2(f)).

The microarchitecture of the lumbar showed the same trend with the tibia after surgery. The tBMD was reduced by 32.5% (*p* < 0.001) in the OVX lumbar and by 42.3% (*p* < 0.001) in the SPX-OVX lumbar relative to Sham, while further decreased by 14.5% (*p* < 0.05) in the SPX-OVX lumbar relative to OVX (Figures 2(g) and 2(h)). The trabecular bone volume (BV/TV) was reduced by 15.3% (*p* < 0.05) in the OVX lumbar and by 32.9% (*p* < 0.01) in the SPX-OVX lumbar relative to Sham, while further decreased by 20.8% (*p* < 0.05) in the SPX-OVX lumbar relative to OVX (Figure 2(i)). The Tb.Th of the lumbar was reduced by12.2% (*p* < 0.05) in the OVX lumbar and by 15.9% (*p* < 0.05) in the SPX-OVX lumbar relative to Sham (Figure 2(j)). The Tb.N was reduced by 20.1% (*p* < 0.05) in the OVX lumbar and by 24.2% (*p* < 0.05) in the SPX-OVX lumbar relative to Sham (Figure 2(k)). The Tb.Sp increased by 21.8% (*p* < 0.05) in the OVX lumbar and by 49.6% (*p* < 0.01) in the SPX-OVX lumbar relative to Sham (Figure 2(l)).

### 3.3. Numbers of Lymphocytes in Bone Marrow Were Altered in OVX and SPX-OVX Rats

**Figure 3:**
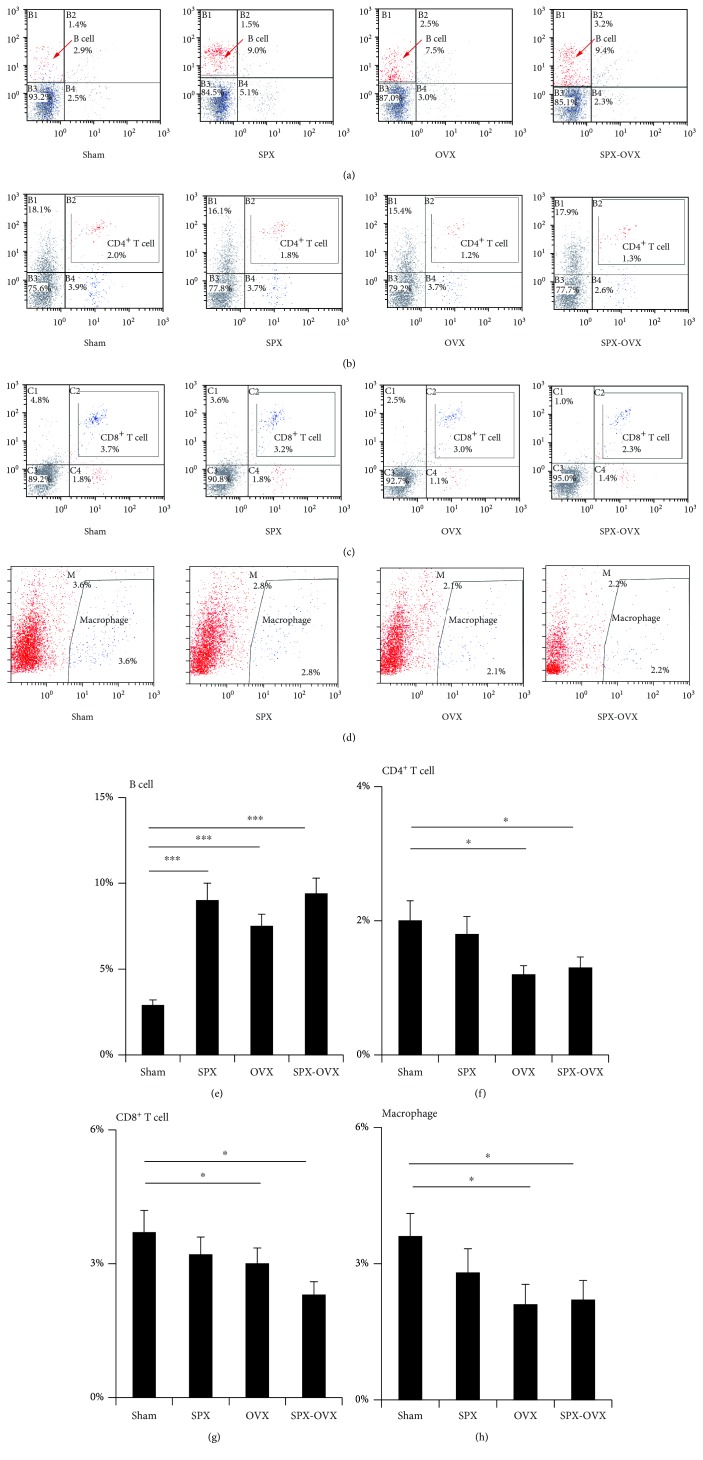
Lymphocytes and macrophages from bone marrow in OVX and SPX-OVX detected by flow cytometry. (a) Flow cytometry of B lymphocytes; (e) B lymphocyte numbers increased in SPX, OVX, and SPX-OVX. (b) Flow cytometry of CD4^+^ T lymphocytes; (f) CD4^+^ T lymphocyte numbers decreased in OVX and SPX-OVX. (c) Flow cytometry of CD8^+^ T lymphocytes; (g) CD8^+^ T lymphocyte numbers decreased in OVX and SPX-OVX. (d) Flow cytometry of macrophages; (h) macrophage numbers decreased in OVX and SPX-OVX. Data are presented as the means ± standard deviations; ^∗^*p* < 0.05 and ^∗∗∗^*p* < 0.001 (*n* = 6/group).

The numbers of lymphocytes in bone marrow changed significantly in OVX and SPX-OVX rats. The B lymphocyte (CD3-CD45RA^+^) in bone marrow increased by 158.6% (*p* < 0.001) in OVX, by 210.3% (*p* < 0.001) in SPX rats, and by 224.1% (*p* < 0.001) in SPX-OVX rats (Figures 3(a) and 3(e)). The CD4^+^ T lymphocytes (CD3-CD4^+^) decreased by 40% (*p* < 0.05) in OVX and by 35% (*p* < 0.05) in OVX-SPX (Figures 3(b) and 3(f)). The CD8^+^ T lymphocytes (CD3-CD8^+^) decreased by 18.9% (*p* < 0.05) in OVX and by 37.8% (*p* < 0.05) in OVX-SPX (Figures 3(c) and 3(g)), while macrophages decreased by 41.7% (*p* < 0.05) in OVX and by 38.9% (*p* < 0.05) in OVX-SPX (Figures 3(d) and 3(h)).

### 3.4. Effect of B Lymphocytes on the Differentiation of BMSCs into Osteoblasts *In Vitro*

**Figure 4:**
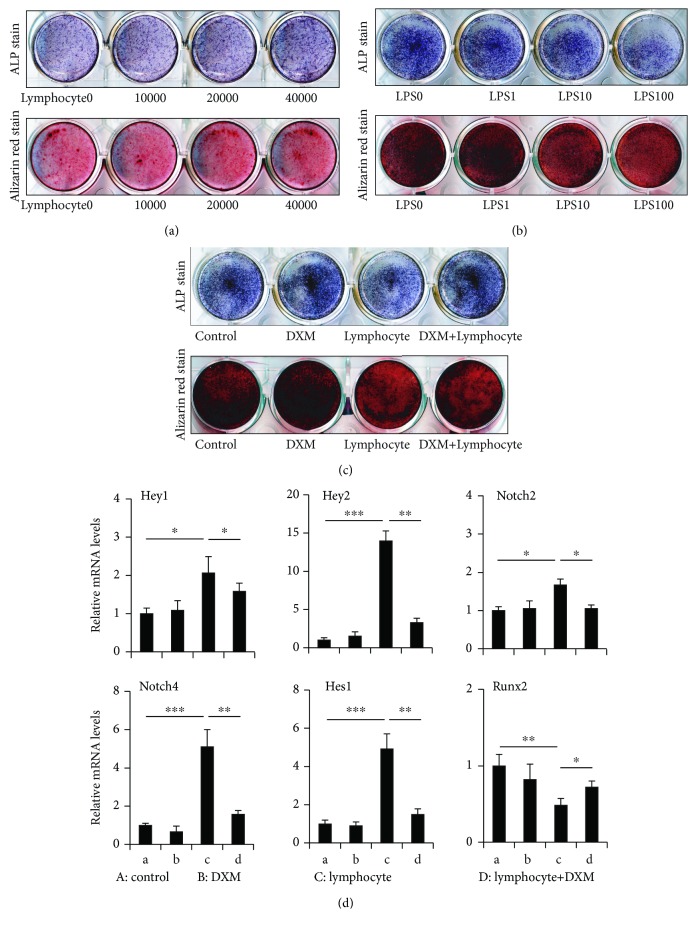
The osteoblastogenesis of BMSCs cocultured with normal B lymphocytes and LPS-pretreated B lymphocytes. (a) ALP and Alizarin red staining of BMSCs cocultured with normal B lymphocytes. (b) ALP and Alizarin red staining of BMSCs cocultured with LPS-pretreated B lymphocytes. (c) ALP and Alizarin red staining of BMSCs cocultured with LPS-pretreated B lymphocytes and dexamethasone. Alizarin red staining: calcified matrix in red and mineralization nodules in the dark. (d) mRNA expression of Hey1, Hey2, Notch2, Notch4, Hes1, and Runx2 in BMSCs. Data are presented as the means ± standard deviations; ^∗^*p* < 0.05, ^∗∗^*p* < 0.01, and ^∗∗∗^*p* < 0.001 (*n* = 6/group).

After being cocultured with normal B lymphocytes, the potential differentiation of BMSCs into osteoblasts did not change obviously (Figure 4(a)). While being cocultured with LPS-pretreated B lymphocytes, the differentiation of BMSCs into osteoblasts decreased. ALP staining showed markedly decreased ALP-positive osteoblasts in cocultures with B lymphocytes from LPS (10, 100) relative to LPS0, and Alizarin red staining showed the same trend (Figure 4(b)).

### 3.5. Effect of DXM on the Differentiation of Cocultured BMSCs into Osteoblasts *In Vitro*

The potential of BMSCs to differentiate into osteoblasts showed no obvious changes in the control and the DXM group. After being cocultured with LPS-pretreated B lymphocytes, the potential of BMSCs to differentiate into osteoblasts clearly decreased, and this would be partly recovered when DXM was used in coculture medium. ALP staining showed markedly decreased ALP-positive osteoblasts in cocultured with LPS-pretreated B lymphocytes relative to control, but the potential of BMSCs to differentiate into osteoblasts clearly was partly recovered if DXM was used in the coculture medium, and Alizarin red staining showed the same trend (Figure 4(c)). The RNA expression of Runx2 decreased significantly in BMSCs after being cocultured with LPS-pretreated B lymphocytes, showing a 51.7% (*p* < 0.01) reduction relative to control and increased by 48.9% (*p* < 0.05) when DXM was used in coculture medium compared to the lymphocyte group (Figure 4(d)). The RNA expression of Hey1, Hey2, Hes1, Notch2, and Notch4 increased in BMSCs after being cocultured with lymphocyte, showing 106.4% (*p* < 0.05), 1299.2% (*p* < 0.001), 391.7% (*p* < 0.001), 67.2%(*p* < 0.05), and 411.1% (*p* < 0.001) relative to control, and all decreased when DXM was used, showing a 23.5% (*p* < 0.05), 76.5% (*p* < 0.01), 69.7% (*p* < 0.01), 36.8% (*p* < 0.05), and 69.3% (*p* < 0.01) reduction compared to the lymphocyte group (Figure 4(d)). The RNA expression showed no obvious changes between control and DXM.

### 3.6. Effect of Notch Inhibitor (DAPT) on the Differentiation of Cocultured BMSCs into Osteoblasts *In Vitro*

**Figure 5:**
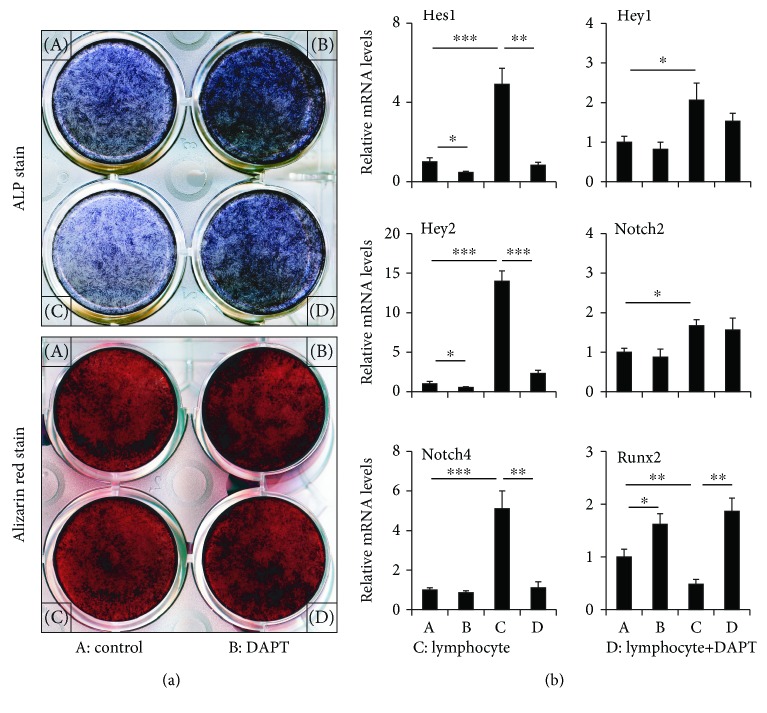
The osteoblastogenesis of BMSCs cocultured with LPS-pretreated B lymphocytes and DAPT. (a) ALP and Alizarin red staining of BMSCs cocultured with LPS-pretreated B lymphocytes and DAPT. Alizarin red staining: calcified matrix in red and mineralization nodules in the dark. (b) mRNA expression of Hey1, Hey2, Notch2, Notch4, Hes1, and Runx2 in BMSCs cocultured with LPS-pretreated B lymphocytes and DAPT. Data are presented as the means ± standard deviations; ^∗^*p* < 0.05, ^∗∗^*p* < 0.01, and ^∗∗∗^*p* < 0.001 (*n* = 6/group).

The potential of BMSCs to differentiate into osteoblasts increased in the DAPT group and decreased in the lymphocyte group. The potential of BMSCs to differentiate into osteoblasts would be partly recovered when DAPT was used in the lymphocyte group. ALP staining showed markedly decreased ALP-positive osteoblasts in cocultures with pretreated B lymphocytes and increased ALP-positive osteoblasts in the DAPT group relative to control. The potential of BMSCs to differentiate into osteoblasts clearly was partly recovered if DAPT was used in the coculture medium, and Alizarin red staining showed the same trend (Figure 5(a)). Runx2 still decreased in BMSCs after being cocultured with B lymphocyte and by 51.7% (*p* < 0.01) in the lymphocyte group compared to control. The Runx2 expression sharply increased by 285.8% (*p* < 0.01) in the lymphocyte+DAPT group compared to the lymphocyte group (Figure 5(b)). The RNA expression of Hey1, Hey2, Hes1, Notch2, and Notch4 increased in BMSCs after being cocultured with B lymphocyte, and the Hes1, Hey2, and Notch4 expression decreased when DAPT was used, showing a 83.1% (*p* < 0.01), 83.5% (*p* < 0.001), and 78.3% (*p* < 0.01) reduction compared to the lymphocyte group (Figure 5(b)). The RNA expression of Runx2 increased by 62.3% (*p* < 0.05) in DAPT relative to control, while the expression of Hes1 and Hey2 decreased by 52.8% (*p* < 0.05) and 46.9% (*p* < 0.05) in DAPT relative to control (Figure 5(b)).

## 4. Discussion

In our study, the osteoporotic phenotypes in OVX rats showed the marked decrease of bone mineral density in the femur, tibia, and lumbar and the significant decrease of the trabecular bone volume, which were worse after spleen removal. All that were not in our expectation of bone loss would be attenuated by splenectomy as like thymectomy. In the research, we found obvious myeloproliferation after surgeries; the medullary cavity of bones was full of lymphocytes. We know that estrogens are potent regulators of B lymphopoiesis at a very early stage [10]; estrogen deficiency may delay the differentiation of B lymphocytes and make them stay at a very early stage. Some studies also show in mice the absolute number of B lymphocyte (defined by the expression of the antigen CD45R/B200) in bone marrow roughly doubles after ovariectomy [4]. In our experiments, we detected the obvious changes in lymphocytes in OVX and SPX-OVX rats, especially in the sharply increased B lymphocytes. The question then arises: why so many B lymphocytes in bone marrow by OVX, even much more by SPX-OVX. Lymphocytes could increase osteoclasts and decrease osteoblasts by producing proinflammatory cytokines in pathological osteoporosis [11–13]. Many researches have illustrated that normal and activated B lymphocytes play different roles in the bone [7, 14]. Normal B lymphocytes could produce osteoprotegerin to increase osteoblastogenesis, and the mice would have osteoporotic phenotype if normal B lymphocytes were knockout [15]. In contrast, the activated B lymphocytes suppressed bone turnover and osteoblastogenesis [7, 16–21]. However, there were few researches on the effects of B lymphocyte on the osteoblastogenesis of BMSCs. Only two studies showed that abnormal lymphocytes inhibited the differentiation of BMSCs into osteoblasts [7, 22]. In our study, we found that LPS-pretreated B lymphocytes inhibited the osteoblastogenesis of BMSCs by coculture, but the normal B lymphocytes did not. It means that only the activated B lymphocytes could induce bone loss, which may be the reason why osteoporosis happened in OVX rats, not in SPX rats, but even much more worse in SPX-OVX rats. Our research showed the activated B lymphocytes will disrupt the bone homeostasis by suppressing the osteoblastogenesis of BMSCs. According to the important relation of bone loss and activated B lymphocytes, it should be given special attention to the therapy of osteoporosis companied with increasing abnormal B lymphocytes.

Dexamethasone, a steroidal anti-inflammatory drug which suppresses lymphocytes, was reported to increase trabecular bone tissue in OVX mice, but nonsteroidal anti-inflammatory drugs which does not suppress lymphocytes did not [7, 23], and the mechanism was not clear. In our study, ALP staining-positive cells increased in the DXM+lymphocyte group relative to the lymphocyte group; a similar trend occurred in Alizarin red staining. It means the potency of differentiation of BMSCs into osteoblasts recovered when activated B lymphocytes were suppressed by dexamethasone. The Notch signaling pathway is conserved and regulates the development of any cells or organisms, of course, which is also deeply involved in the development of B lymphocytes and bone [24, 25]. Recent studies showed that Notch signaling enhanced B lymphocyte differentiation, proliferation, and T-dependent immune response [26]. Researches about bone showed that activation of Notch signaling arrested pluripotent precursors to the osteoblastic lineage, but inactivation of Csl/Rbpj*κ* restores normal osteoblastic differentiation [24, 27, 28]. Studies from different fields seem to demonstrate that the activated Notch signaling would play entirely different roles on different cell developments. As we all know, the Hes family and the Hey family are target genes of notch intracellular domain in the nucleus. Overexpression of Hes1 causes osteopenia in female mice, and inactivation of Hes1 in osteoblasts increases trabecular bone volume in male mice [29]. Taken together, these researches highlight that activated Notch signaling is essential in regulating B lymphocyte survival, activation, proliferation, and differentiation. Meanwhile, the osteoblastogenesis of BMSCs will be suppressed when Notch signaling is activated. Notch signaling play an unexpected role in the balance of homeostasis between the development of B lymphocytes and osteoblasts differentiated from BMSCs. In our study, we found that the mRNA expression of Notch2, Notch4, Hes1, Hey1, and Hey2 increased in BMSCs cocultured with pretreated B lymphocytes, especially Notch4, Hes1, and Hey2, while the high expression will turn down when dexamethasone was used in the coculture system. This study showed that activated B lymphocytes would activate Notch signaling in BMSCs, while dexamethasone can weaken this effect by suppressing the activated B lymphocytes. As we all know, the transactivation of Runx2 is necessary for osteoblast differentiation of BMSCs, and it is also a target gene of notch intracellular domain [27]. So we also detected that the expression of Runx2 decreased in BMSCs cocultured with activated B lymphocytes, and this effect would partly restore when dexamethasone was used. Meanwhile, DAPT (an inhibitor of Notch signaling) was used in the coculture system; the suppression of osteoblastogenesis of BMSCs was also restored by inhibiting the activated Notch signaling of BMSCs. The expression of Notch4, Hes1, and Hey2 was sharply decreased, and the expression of Runx2 increased when DAPT was used in the coculture system.

## 5. Conclusions

This study revealed a role of B lymphocytes in the osteoblastogenesis of BMSCs in ovariectomized and splenectomized-ovariectomy rats. The much more serious osteoporosis accompanied with increasing B lymphocytes in bone marrow by splenectomy which was opposite to the outcomes of thymectomy and which suggested the balance of the immune system, especially among immune organs, contributes to the maintenance of skeletal homeostasis in estrogen deficiency. Meanwhile, Notch signaling is activated in BMSCs when cocultured with LPS-pretreated B lymphocytes. The expression of Notch2, Notch4, Hes1, Hey1, and Hey2 increased in cocultured BMSCs and decreased when dexamethasone (suppress the lymphocytes) or DAPT (inhibitor of Notch signaling) was in the coculture system. It means that the activated B lymphocytes would suppress the osteoblastogenesis of BMSCs by activating the Notch signaling, and the osteoblastogenesis of BMSCs would partly restore when B lymphocytes were suppressed or Notch signaling was inactivated. It should be taken into attention that inhibitors of Notch signaling may play a promising role in osteoporosis accompanied with abnormal B lymphocyte differentiation.

## Data Availability

The data used to support the findings of this study are available from the corresponding authors upon request.

## Abbreviations

BMSCs: Bone mesenchymal stem cells
Runx2: Runt-related transcription factor 2
ALP: Alkaline phosphatase
tBMD: Trabecular bone mineral density
BV/TV: Bone volume fraction, ratio of the segmented bone volume to the total volume of the region of the region of interest
Tb/Th: Trabecular thickness, mean thickness of trabeculae
Tb.Sp: Trabecular separation, mean distance between trabeculae
Tb.N: Trabecular number, measure of the average number of trabeculae per unit length.
